# The Effect of High Dose Folic Acid throughout Pregnancy on Homocysteine (Hcy) Concentration and Pre-Eclampsia: A Randomized Clinical Trial

**DOI:** 10.1371/journal.pone.0154400

**Published:** 2016-05-11

**Authors:** Manizheh Sayyah-Melli, Amir Ghorbanihaghjo, Mahasti Alizadeh, Maryamalsadat Kazemi-Shishvan, Morteza Ghojazadeh, Sanam Bidadi

**Affiliations:** 1 Drug Applied Research Center (DARC), Tabriz University of Medical Sciences, Postal address, 5138665793, Tabriz, Iran; 2 Biotechnology Research Center, Tabriz University of Medical Sciences, Tabriz, Iran; 3 Social Determinants of Health Research Center, Tabriz University of Medical Sciences, Tabriz, Iran; 4 Department of Community Medicine, Faculty of Medicine, Tabriz University of Medical Sciences, Tabriz, Iran; 5 Women's Reproductive Health Research Center, Tabriz University of Medical Sciences, Tabriz, Iran; University of Ottawa, CANADA

## Abstract

Pre-eclampsia is a pregnancy-related multi-systemic hypertensive disorder and affects at least 5% of pregnancies. This randomized clinical trial aimed at assessing the effect of low doses and high doses of folic acid on homocysteine (Hcy) levels, blood pressure, urea, creatinine and neonatal outcome. A randomized clinical trial was done at Alzahra Teaching Hospital, Tabriz University of Medical Sciences from April 2008 to March 2013. Four-hundred and sixty nulliparous pregnant women were randomly assigned into two groups. Group 1 (n = 230) received 0.5 mg of folic acid and group 2 (n = 230) received 5 mg of folic acid per daily. They were followed until delivery. Blood pressure and laboratory changes, including plasma Hcy levels, were measured and compared between the groups. Homocysteine concentrations were significantly higher at the time of delivery in group 1 (13.17±3.89 μmol/l) than in group 2 (10.31±3.54, μmol/l) (p<0.001). No statistically significant differences were observed in systolic and diastolic blood pressure (p = 0.84 and 0.15, respectively). Birth weight was significantly higher in group 2 (p = 0.031) and early abortion was significantly higher in group 1 than group 2 (p = 0.001). This study has provided evidence that a high dosage of folic acid supplements throughout pregnancy reduces Hcy concentrations at the time of delivery.

***Trial Registration*:** Iranian Registry of Clinical Trials IRCT201402175283N9

## Introduction

One of the main goals of prenatal care is control of hypertension disorders and pre-eclampsia. Pre-eclampsia is one of the unsolved puzzles in medicine with high prevalence and maternal mortality, especially in low- and middle-income countries [1]. One of the main hypotheses associated with the cause of pre-eclampsia is placental insufficiency and its vascular discrepancy, which is caused by increasing levels of homocysteine (Hcy) [2]. Homocysteine is a sulfur-containing amino acid metabolized through remission of methionine, which is one of the two pathways requiring folic acid (Methyltetrahydrofolate pathway) [3]. Decreasing folate levels lead to lower erythrocyte folate levels, an increase in Hcy concentration and variations in other fast-growing tissues [4]. These problems may cause a vaso-occlusive effect on the placenta, neural tube defects, recurrent abortion and pre-eclampsia late in pregnancy [5]. It has been shown that the risk of pre-eclampsia in pregnant women with hyperhomocysteinemia and low folate status increased several times compared to the controls [6]. One study showed that taking high doses of folic acid (3–9 mg daily) reduced the rate of preterm labor and early onset of pre-eclampsia [7]. In another study, folic acid intake early in the second trimester reduced the risk of pre-eclampsia [8]. There was no difference in the rate of pregnancy complications or pre-eclampsia between taking 200 μg, 400 μg and 5 mg of folic acid per day in another study [9, 10]. In recent years, the role of vascular dysfunction and increased Hcy levels have been taken into consideration, and high Hcy levels are considered a predicting factor for pre-eclampsia [11]. Despite some strategies considered effective in predicting those who might be benefited from treatment, so far no valid method is known for identifying individuals at risk and the actual mechanism is still unknown [1]. Supplementation of 4 mg of folic acid throughout pregnancy is considered a new prevention strategy for pre-eclampsia. Daily supplementation with 4 mg of folic acid starting in early pregnancy (8 to 16 weeks of gestation) until delivery has been effective in preventing pre-eclampsia [12]. Other observational studies have been conducted to show whether folic acid supplementation during pregnancy can reduce the risk of pre-eclampsia [13].

These findings indicate that high doses of folic acid (much higher than the amount received from food or what is usually taken during pregnancy) and/or long duration of use may be required during pregnancy for the prevention of pre-eclampsia. Further studies are needed to specify whether a Hcy metabolism disorder is the main cause or increased Hcy levels are a secondary cause of pre-eclampsia/eclampsia during pregnancy and whether it is necessary to maintain the high level of folate or not. There is no definitive known cause of pre-eclampsia and there is no definitive way to identify the individuals at risk. It occurs in women with first or multiple pregnancies and is characterized by new onset hypertension and proteinuria [14]. Preeclampsia has several different underlying pathologies and pathologic phenotypes. So identifying women at risk from low-risk individuals is very difficult from clinical characteristics and biochemical markers in first-trimester women that would possibly predict the subsequent development of preeclampsia. [15].According to the results of various studies on early onset pre-eclampsia and hyperhomocysteinemia, the rationale for this study was to investigate the benefits of high doses of folic acid supplementation compared to low doses on the level of Hcy and for the prevention of pre-eclampsia/eclampsia.

Our previous study was carried out among women randomized by alternate allocation to a group receiving high doses (5 mg/day) of folic acid compared with low doses (0.5 mg/day) of folic acid throughout pregnancy; however, the findings were based on a small sample size [16].

This study extends the previous analysis by including data from a larger sample size of healthy pregnant women to investigate whether high doses of folic acid supplementation (5 mg/day), compared to low doses (0.5 mg/day), can reduce pre-eclampsia and eclampsia by reducing Hcy levels throughout pregnancy.

## Materials and Methods

### Ethics statement

The study was approved by the pharmaceutical research center of Tabriz University of Medical Sciences (TUOMS) and it was registered in the Iranian Clinical Trials registry (IRCT201402175283N9). The TUOMS’ ethical committee approved the use of the regimens. All participants provided written informed consent themselves.

### Study design and intervention

A randomized controlled trial of nulliparous pregnant women was conducted in an antenatal outpatient clinic of Alzahra Hospital, a tertiary care university hospital in Tabriz, Iran, from May 2008 to April 2013.

The trial was registered retrospectively due to the policy of registration at the time of the proposal approval. We confirmed that all related clinical trials are registered in clinical trial registries. A summarized version of the clinical trial protocol is available as S1 Appendix. Only healthy mothers between 20–30 years of age with a singleton pregnancy coming for prenatal care were studied. Participants with any history of medical problems and hyperemesis gravidarum were excluded. Mothers who were taking supplements other than ferrous sulfate, vitamin B6 and calcium, having an abortion and not coming to the assigned teaching hospital for delivery were also excluded. This study was an extension of our previous study and the sample size was determined by the results of that study [16], where the level of Hcy (μmol/l) within each subject group was normally distributed with a standard deviation of 3.85. If the true difference in the low-dose and high-dose means is 1, we would need to study 230 low-dose subjects and 230 high-dose subjects to be able to reject the null hypothesis that the population means of the low-dose and high-dose groups are equal with probability (power) 0.8. The type I error associated with the test of this null hypothesis is 0.05.

### Recruitment and randomization

A random allocation sequence was generated based on the type of intervention using computer-generated random numbers by a person from the research team who was not involved in recruiting and assigning of the participants. Using a simple random allocation scheme, each participant got a number and was assigned to study groups with equal probability. For the allocation sequence concealment from those assigning participants to intervention groups, the random numbers were printed out, each sheet was placed one by one into an envelope and the envelopes were opened in sequence by an investigator at the moment of assignment. The participants and investigator were aware of the type of intervention.

### Data collection and follow-up

**Fig 1:**
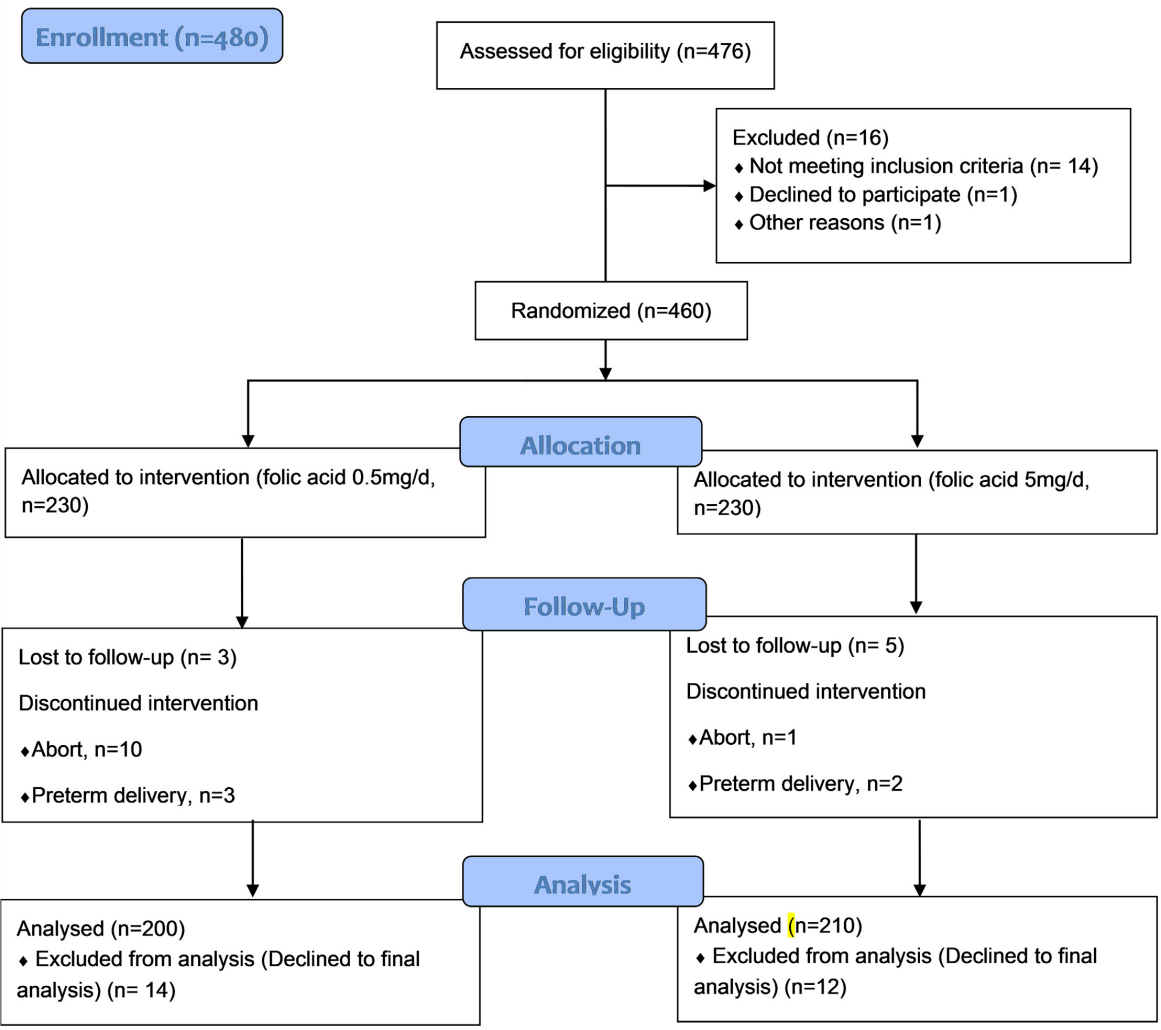
The CONSORT Flow Diagram of participants.

The participants were given folic acid (manufactured by Maad Pharmaceutical Co.) daily from early pregnancy until delivery (0.5 mg/day in group 1 and 5 mg/day in group 2). The lab specialist and analyst were unaware of the treatment assignments and were blinded to sample provenance based on the response. Before the supplementation of folic acid, the plasma concentrations of urea and uric acid, plasma level of creatinine, lactate dehydrogenase (LDH), platelet count, urine creatinine and urine protein were measured enzymatically and by radioimmunoassay at the first visit. These were measured again at delivery. The blood samples for Hcy levels were collected in tubes containing Ethylenediaminetetraacetic acid (EDTA) and were centrifuged within one hour. The plasma was stored at 2–8°C until the assay was performed within 72 hours after collection. Plasma Hcy levels were measured before starting the folic acid intake and also before labor with enzymatic and radioimmunoassay as well (Axis Hcy Enzyme Immunoassay [EIA] Technology) (Hcy EIA; FHVY100, Axis-Shield, UK). The band Hcy (visible form) was reduced to the free form, which was enzymatically converted to S-adenosy-L-Hcy (SAH). Blood pressure was measured at the beginning of pregnancy monthly until 28 weeks of pregnancy, every two weeks until week 36 of pregnancy and then weekly in the last month of pregnancy. Blood pressure more than or equal to 140/90 mm Hg was considered gestational hypertension, which was measured on two occasions at least 6 hours apart. Blood pressure higher than 160/110, along with other symptoms such as proteinuria, blurry vision, epigastric pain, headache and edema without and with seizures, was considered severe pre-eclampsia and eclampsia, respectively. All mothers took 1g of calcium and 60 mg of ferrous sulfate daily from week 14 of pregnancy until delivery (Fig 1).

### Statistical Analysis

Continuous’ variables are presented as means (SD). The independent samples t-test was used to compare mean of quantitative variables between two groups. We tested the mixed model using covariance structure AR (1) and REML (Restricted maximum likelihood) estimation method to compare means of post-intervention continuous outcomes between two groups. Post hoc comparison performed using the Tukey test to compare quantitative variables in time before and after intervention.Within-group (before/after intervention) differences were assessed using paired samples t-test. Categorical data were reported as frequencies (percentages) and were tested by chi square or Fisher's exact test. The normality of the variables distribution was tested by the Shapiro-Wilk test. Statistical analyses were performed using SPSS 17.0 software. P-values less than 0.05 were considered statistically significant.

## Results and Discussion

Originally, 480 women were recruited, of whom 476 were assessed for eligibility. A total of 460 women met the eligibility criteria and were randomized into the low- or high-dose folic acid groups. Eleven patients aborted early in the first trimester and 40 patients they were excluded from the study the final analysis because they did not return for follow-up during pregnancy or delivery or for other reasons. A total of 410 women were analyzed for the primary outcomes as shown in Fig 1, which is participants’ CONSORT Flow Diagram. Some demographic characteristics of the studied population are shown in “Table 1”. The baseline characteristics were analyzed and found to be similar between groups and are displayed in “Table 1”.

**Table 1:** Demographic variables in pregnant women who received low doses and high doses of folic acid supplementation.

**Variables**	**Group 1 (Low dose)(N = 200)**	**Group 2 (High dose)(N = 210)**	**P-value**
Age (years)	25.20(3.36)	25.17(3.75)	0.93
Height _cm_	1.62(0.05)	1.63 (0.06)	0.25
Weight_(kg)_	65.96 (9.86)	65.36 (8.28)	0.51
^c^BMI_(kg/m2)_	25.07(3.42)	24.68(2.91)	0.21

Notes: All values are mean (SD). Significances are based on independent samples t-test, ^c^BMI: body mass index.

The results showed that Hcy concentrations were significantly higher at the time of delivery in group 1 (13.17±3.89 μmol/l) than in group 2 (10.31±3.54, μmol/l) (p<0.001) “Table 2”. The changes in other laboratory parameters before and after receiving low dose or high dose of folic acid supplementation were also shown in “Table 2”.

**Table 2:** Laboratory parameters of pregnant women before and after receiving low dose or high dose of folic acid supplementation.

Parameters	Low dose (n = 200)	High dose (n = 210)
Hcy (mcmol/l)Baseline	10.31(3.54)	13.17(3.89)
Hcy (mcmol/l)Endpoint	8.46(3.35)	7.20(3.35)
Plasma creatinine (mg/dl) Baseline	0.74(0.10)	0.71(0.10)
Plasma creatinine (mg/dl) Endpoint	0.70(0.09)	0.67(0.08)
Urine creatinine (mg/dl) Baseline	0.91(0.18)	0.91(0.19)
Urine creatinine (mg/dl) Endpoint	0.88(0.15)	0.88(0.53)
LDH (U/L)Baseline	292.84(86.65)	292.10(82.80)
LDH (U/L)Endpoint	293.23(90.38)	307.72(84.12)
Urea (mg/dl)Baseline	23.08(6.33)	21.20(7.83)
Urea (mg/dl)Endpoint	21.81(6.17)	21.05(6.36)
Uric acid (mg/dl) Baseline	3.84(0.72)	3.69(0.75)
Uric acid (mg/dl)Endpoint	4.30(1.15)	3.94(0.70)
BP (systolic) (mmHg)Baseline	118.92 (8.11)	116.31 (9.52)
BP (systolic) (mmHg)Endpoint	120.59 (10.61)	117.36 (9.51)
BP (diastolic) (mmHg) Baseline	76.50 (5.87)	74.57 (7.53)
BP (diastolic) (mmHg) Endpoint	78.07 (7.18)	74.73 (7.44)

All values are mean (SD)

There were no adverse effects in each group. No cases of pre-eclampsia or eclampsia were observed in the two groups. “Table 3” shows the results in adjusting for plasma creatinine, urine creatinine, LDH, urea, uric acid, blood pressure (systolic), and blood pressure (diastolic). In low dose group significant difference was observed between sub groups before and after intervention. The post hoc test showed that there was significant difference among four sub group low dose-before, low dose-after, high dose-before and high dose-after (confidence intervals did not overlap). In low dose group variation between before and after intervention was equal to 2.14 and this value in high dose group was equal to 6.48.

**Table 3:** Results of Generalized linear mixed model with Fixed Effects.

**Variables**	**F**	**P-value**
Plasma Cr	2.573	.109
Urine Cr	7.408	.007
LDH	.005	.943
Urea	31.916	<0.001
UricAcid	3.353	.068
BP(Sys)	.037	.848
BP(Dias)	1.990	.159
Group * Time	237.360	<0.001

* Results in adjusting for Plasma creatinine, Urine creatinine

LDH, Urea, Uric acid, Blood pressure (Systolic), Blood pressure (Diastolic)

Therefore, homocysteine changes between high dose groups were significantly higher than low dose group “Table 4”.

**Table 4:** Results of Group * Time interaction*.

Group	Adjusted Mean	95% Confidence Interval	
		Lower Bound	Upper Bound
Low dose(Before)	11.085**	10.445	11.726
Low dose(After)	8.940**	8.332	9.549
High dose(Before)	14.111**	13.555	14.666
High dose(After)	7.631**	7.073	8.188

*Dependent Variable: Hcy.

** Covariates appearing in the model are evaluated at the following values: PlasmaCr = .7239, UrineCr = .8948, LDH = 306.7485, Urea = 24.3148, UricAcid = 3.7708, BP (Sys) = 119.4711, BP (Dias) = 75.9461.

Results of neonatal outcomes showed no significant differences between groups except for birth weight which was significantly higher in group 2 (p<0.03) “Table 5”.Early abortion was significantly higher in group 1 than in group 2(RR = 0.95, 95%CI (0.92–0.98) “Table 6”. Statistical analyses showed a significant difference between low and high doses of folic acid on Hcy before and after intervention (*p*<0.0001).

**Table 5:** Results of neonatal outcomes in pregnant women who received low doses and high doses of folic acid.

Variables	Group 1 (Low dose)	Group 2 (High dose)	P-value
Birth weight (gr)	3366.12(421.39)	3456.39(410.30)	0.031*
Neonatal height (cm)	50.67(1.95)	51.03(3.78)	0.243
Head circumference (cm)	35.90(0.52)	36.10(1.73)	0.232
Hospital stay (days)	2.12(0.52)	2.09(0.29	0.549
Apgar (1 min)	8.89(0.37)	8.90(0.42)	0.956
Apgar (5 min)	10.27(2.56)	10.00(0.00)	0.320

Notes: All values are mean (SD). Significances are based on independent samples t-test.

*p-value<0.05.

**Table 6:** Pregnancy outcome in pregnant women who received low doses and high doses of folic acid.

Variables	Low dose(N = 200)		High dose(N = 210)		P-value	RR	%95 CI
	Yes	No	Yes	No			
PROM^a^	31(15.5)	169(84.5)	37(17.6)	173(82.4)	0.564	0.88	(0.56–1.36)
Early abortion	10(5)	190 (95)	1(0.5)	209(99.5)	0.005	0.95	(0.92–0.98)
Late abortion	0(0)	200(100)	1(0.9)	209(99.5)	-	-	-
Gestational hypertension	1(0.9)	199 (99.1)	0(0)	210(100)	-	-	-
Eclampsia	0(0)	200	0(0)	210(100)	-	-	-
Pre-eclampsia	0(0)	200	0(0)	210(100)	-	-	-
Headache	8(4)	192(96)	3(1.4)	207(98.6)	0.107	2.80	(0.75–10.4)
Blurred vision	1(0.5)	199(99.5)	0(0)	210(100)	-	-	-
Heart burn	1(0.5)	199(99.5)	0(0)	210(100)	-	-	-

Notes: All values are frequencies (percentages). Significances are based chi square test.

^a^PROM: premature rupture of membrane.

## Discussion

In our previous study, we reported the effectiveness of a high dose of folic acid throughout pregnancy on homocysteine levels [16]. This study confirms those observations using a larger sample size. In this study, we demonstrated that daily folic acid intake with either doses of 0.5 mg or 5 mg throughout pregnancy significantly reduced the plasma levels of Hcy. While the baseline Hcy levels between groups show a statistically significant difference at the first trimester, that is not clinically important and they were both at normal range “Table 2”. Any differences at baseline were due to chance. The ideal level of homocysteine is not clear but according to the literature its normal levels are differ from 2.2 to 13.2 μmol/l and 4–17.2 μmol/l [17]. The level of Hcy was reduced significantly at term in group 2 who were taking higher doses of folic acid “Table 2”. No cases of pre-eclampsia or eclampsia was reported. However, the relative risk of headache was 2 (95%CI 0.75–10.4) and it was not significant, the cause of the wide CI is the small number of patients with headache. Results in adjusting for plasma creatinine, urine creatinine, lactate dehydrogenase (LDH), urea, uric acid, systolic blood pressure and diastolic blood pressure show that it is possible thefolic acid dose and related changes in metabolism are responsible for some or all of the observed differences “Table 3”.

Research studies have been conducted to understand the metabolism of Hcy in the physiology of pregnancy. The causal relationship has not been entirely proven [18]. In recent years, the role of vascular disorders and increased levels of Hcy have been taken into consideration [19, 20]. Several studies have demonstrated an association between Hcy levels and pre-eclampsia, but this association has not been stated with certainty [6, 10, 21]. It is extremely important to avoid development of pre-eclampsiaand eclampsia because of the association with high prevalence of pregnancy complications and maternal mortality. Therefore, any safe intervention is essential. Currently, clinical trials are ongoing to determine whether lowering Hcy with folic acid intake is associated with improved clinical prognosis or not. Şanlıkan et al. found that the levels of Hcy in the plasma of patients with pre-eclampsia, irrespective of the severity, were high [22]. The results of other studies show that factors such as race and genetics may have a predicting role [23].

Folate has been recognized as an effective Hcy-lowering agent [24]. Folate works directly or indirectly, but the final results improve performance of endothelium in both placental and systemic perfusion [25]. Although clinical studies have shown links between them, other researchers have not found it to be a useful determinant [26, 27]. The results of our study showed a greater risk for early abortion in group 1 than in group 2”Table 6”. According to studies of high-dose folic acid supplementation in epileptic women undergoing antiepileptic therapies, the results showed that the rate of abortion is reduced in these patients [28]. This study and ours have shown an effective role for high doses of folic acid in the prevention of abortion. There was a significant difference between birth weights in the two groups, which were significantly higher in group 2 (p = 0.031) “Table 5”, receiving 5 mg of folic acid throughout pregnancy. In contrast to our study, Czeizel and Bánhidy could not show the efficacy of 0.8 mg of folic acid-containing multivitamin used in the periconceptional period on the reduction of birth weight and gestational age at delivery [29]. The findings of these authors appear to be reasonable, because in their study pregnant women were given supplementation from the 12th preconception week until the 12th postconception week, whereas major fetal growth occurs after the first trimester, mainly during the third trimester. However, in the study of Timmermans and colleagues, periconceptional folic acid supplementation was effective in reducing low birth weight [30]. Pre-eclampsia is a pregnancy-related disorder. Taking multivitamins containing folic acid may considerably reduce the risk of pregnancy-induced hypertension or pre-eclampsia [4] as shown in our study. There is no definitive way to identify the individuals at risk. Despite the beneficial effects of prescribing folic acid as a supplement for the prevention of developmental abnormalities and age-related diseases, its role in the prevention of pre-eclampsia is still less clear [23].

In most studies of pre-eclampsia, the influence of different doses of folic acid along with vitamins B6 and B12 on the amount of plasma Hcy was examined [4, 26, 31, 32]. In the study of Wen and colleagues,early second trimester folic acid supplementation increased serum folate, decreased plasma Hcy and reduced risk of pre-eclampsia [8]. Although in some studies high doses of folic acid have been prescribed safely throughout pregnancy for reduction of pregnancy complications [7, 33], the effects of high-dose folate on both the mother and the offspring are still being investigated. The Hordaland Hcy study also showed that high Hcy concentrations were associated with risks of pre-eclampsia, premature delivery and low birth weight [18]. In comparison with our study, Fernandez et al. failed to find a significant difference between the concentration of Hcy and the prevalence of hyperhomocysteinemia in various stages of pregnancy. There was no significant difference after three months of taking 1 mg/day of folic acid between the normal mothers and those who developed pre-eclampsia. In their study, the sample size was small, and the duration and amount of folic acid intake were less than in ours [21]. Acilmis et al. discovered a relationship between pre-eclampsia and high levels of Hcy, but not folic acid deficiency [34]. Salehi-PourMehr et al. alsofound an association between hyperhomocysteinemia and pre-eclampsia/eclampsia [35].

In our study, the higher dosage of folic acid decreased plasma Hcy levels more. So that further clinical trials are necessary to determine the result of decreased levels of Hcy on future maternal and fetal outcomes. Ultimately, for a better understanding of this relationship, observations are needed to corroborate these findings and further explore the benefit (if any). Recent concerns about high doses of folic acid have been examined in a systematic review study and through meta-analysis on the effect of folate on the development of cancer, but the results were quite inconsistent with each other. There was no difference in the mortality rate of the case and control groups. Based on experimental studies, folic acid deficiency may stimulate early stages of carcinogenesis and high dosages of prescribed folic acid thereafter can cause cancer cells to grow. Based on these researchers’ opinions, more studies are need to be conducted in different geographic regions to give a clearer statement [36, 37]. In contrast, in a meta-analyses of data on 50 000 individuals, Vollset et al. found no significant effect of folic acid supplementation on the incidence of cancer of the large intestine, prostate, lung, breast or any other specific site [38]. In addition, observations on women with higher plasma folate levels and HPV 16 methylation showed that folic acid played a critical role in lowering the HPV 16 methylation-associated risk of developing higher grades of CIN [39].

Limitations of this study were the large normal range of Hcy levels and differences in these levels at the beginning of the study. Other limitations were included not blinding the participants/researchers which might have had the effect on the results, and biased estimates of treatment effects. But this effect is not significant because most of the outcomes are objective and the placebo effect and measurement bias is not so noteworthy. Moreover, not all patients referred to the university hospital at the time of delivery.

According to the results of our study, high dose of folic acid supplement throughout pregnancy reduce Hcy concentrations at the time of delivery.

## Conclusion

The results show that folic acid with a dose of 5 mg/day accompanies with reduction in the levels of plasma Hcy and improvements in other laboratory findings. Based on the results of this study, 5 mg/day of folic acid is recommended during the whole pregnancy to lower the Hcy levels, but in this respect, more control studies need to be conducted to clarify the role of increased Hcy in pregnant women who subsequently develop pre-eclampsia/eclampsia in addition to elucidate the long-term effects of high-dose folic acid supplementation throughout pregnancy on offspring.

## Supporting Information

S1 Appendix Data file containing Clinical trial protocol “The effect of prenatal administration of high-dose and low-dose folic acid on maternal plasma homocysteine concentration and its relationship with pre-eclampsia.” (DOCX)

S2 Appendix CONSORT checklist. (DOC)

S1 Data Dataset underlying the findings (SPSS file). (SAV)

## References

[1] Payne BA, Hutcheon JA, Ansermino JM, Hall DR, Bhutta ZA, Bhutta SZ, et al A Risk Prediction Model for the Assessment and Triage of Women with Hypertensive Disorders of Pregnancy in Low-Resourced Settings: The miniPIERS (Pre-eclampsia Integrated Estimate of Risk) Multi-country Prospective Cohort Study. PLoS Med. 2014;11: e1001589 10.1371/journal.pmed.1001589 24465185 PMC3897359

[2] Laskowska M, Laskowska K, Terbosh M, Oleszczuk J. A comparison of maternal plasma levels of endothelial nitric oxide synthase, asymmetric dimethylarginine, and Hcy in normal and preeclamptic pregnancies.Med Sci Monit. 2013;19: 430–437. 10.12659/MSM.883932 23739294 PMC3675838

[3] Guilland JC, Favier A, Potier de Courcy G, Galan P, Hercberg S. Hyperhomocysteinemia: an independent risk factor or a simple marker of vascular disease?. Pathol Biol (Paris). 2003;51: 101–110. 12801808 10.1016/s0369-8114(03)00104-4

[4] Yanez P, Vásquez CJ, Rodas L, Durán A, Chedraui P, Liem KH, et al Erythrocyte folate content and plasma folic acid and Hcy levels in preeclamptic primigravidae teenagers living at high altitude. Arch Gynecol Obstet. 2013;288: 1011–1015. 10.1007/s00404-013-2851-7 23609037

[5] Ray JG, Laskin CA. Folic acid and Hcy metabolic defects and the risk of placental abruption, pre-eclampsia and spontaneous pregnancy loss: a systematic review. Placenta. 1999;20: 519–529. 10452905 10.1053/plac.1999.0417

[6] López-Quesada E1, Vilaseca MA, Lailla JM. Plasma total Hcy in uncomplicated pregnancy and in preeclampsia. Eur J Obstet Gynecol Reprod Biol. 2003;108: 45–49. 12694969 10.1016/s0301-2115(02)00367-6

[7] Bánhidy F, Dakhlaoui A, Dudás I, Czeizel AE. Birth Outcomes of Newborns after Folic Acid Supplementation in Pregnant Women with Early and Late Pre-Eclampsia: A Population-Based Study. Adv Prev Med. 2011. 10.4061/2011/127369 PMC3168906 21991429

[8] Wen SW, Chen XK, Rodger M, White RR, Yang Q, Smith GN, et al Folic acid supplementation in early second trimester and the risk of pre-eclampsia. Am J Obstet Gynecol. 2008;198: 45.e1–7. 18166303 10.1016/j.ajog.2007.06.067

[9] Charles DH, Ness AR, Campbell D, Smith GD, Whitley E, Hall MH. Folic acid supplements in pregnancy and birth outcome: re-analysis of a large randomised controlled trial and update of Cochrane review. Paediatr Perinat Epidemiol. 2005;19:112–124. 15787886 10.1111/j.1365-3016.2005.00633.x

[10] Li Z, Ye R, Zhang L, Li H, Liu J, Ren A. Folic acid supplementation during early pregnancy and the risk of gestational hypertension and pre-eclmpsia. Hypertension. 2013; 61:873–879. 10.1161/HYPERTENSIONAHA.111.00230 23399716

[11] Kajdy A1, Niemiec T. Hcy metabolism disorders as a potential predictor of preeclamsia. Ginekol Pol. 2008; 79:775–779. 19140501

[12] Wen SW, Champagne J, Rennicks White R, Coyle D, Fraser W, Smith G, et al Effect of folic acid supplementation in pregnancy on preeclampsia: the folic acid clinical trial study.J Pregnancy. 2013; 2013:294312 10.1155/2013/294312 24349782 PMC3852577

[13] Wen SW, Champagne J, Rennicks White R, Walker M. Effect of folic acid supplementation in pregnancy on preeclampsia- Folic acid clinical trial (FACT). Pregnancy Hypertens. 2012;2(3):198. 10.1016/j.preghy.2012.04.041 26105254

[14] Al-Jameil N, Aziz Khan F, Fareed Khan M, Tabassum H. A brief overview of preeclampsia. J Clin Med Res. 2014;6(1):1–7. 10.4021/jocmr1682w 24400024 PMC3881982

[15] Myatt L, Clifton RG, Roberts JM, Spong CY, Hauth JC, Varner MW, et al First-trimester prediction of preeclampsia in nulliparous women at low risk. Obstet Gynecol. 2012; 119(6):1234–42. 10.1097/AOG.0b013e3182571669 22617589 PMC3360523

[16] Manizheh SM, Mandana S, Hassan A, Amir GH, Mahlisha KS, Morteza G. Comparison study on the effect of prenatal administration of high dose and low dose folic acid. Saudi Med J. 2009; 30:88–97. 19139780

[17] Loehrer FM, Schwab R, Angst CP, Haefeli WE, Fowler B. Influence of oral S-adenosylmethionine on plasma 5-methyltetrahydrofolate, S-adenosylhomocysteine, homocysteine and methionine in healthy humans. J Pharmacol Exp Ther. 1997;282(2):845–50. 9262350

[18] Vollset SE, Refsum H, Irgens LM, Emblem BM, Tverdal A, Gjessing HK, et al Plasma total Hcy, pregnancy complications, and adverse pregnancy outcomes: the Hordaland Hcy study. Am J Clin Nutr. 2000;71: 962–968. 10731504 10.1093/ajcn/71.4.962

[19] Schaffer A, Verdoia M, Cassetti E, Marino P, Suryapranata H, De Luca G; et al Relationship between homocysteine and coronary artery disease. Results from a large prospective cohort study. Thromb Res. 2014;134(2):288–93. 10.1016/j.thromres.2014.05.025 24928335

[20] Dinavahi R, Falkner B. Relationship of homocysteine with cardiovascular disease and blood pressure. J Clin Hypertens (Greenwich). 2004;6(9):494–8. 15365276 10.1111/j.1524-6175.2004.03643.x PMC8109446

[21] Fernandez M, Fernandez G, Diez-Ewald M, Torres E, Vizcaino G, Fernandez N, et al Plasma Hcy concentration and its relationship with the development of preeclampsia. Effect of prenatal administration of folic acid. Invest Clin. 2005;46: 187–195. 16001750

[22] Şanlıkan F, Tufan F, Göçmen A, Kabadayı C, Şengül E.The evaluation of homocysteine level in patients with preeclampsia. Ginekol Pol. 2015;86(4):287–91. 26117988 10.17772/gp/2075

[23] Ji Y, Kong X, Wang G, Hong X, Xu X, Chen Z, et al Distribution and determinants of plasma homocysteine levels in rural Chinese twins across the lifespan. Nutrients. 2014; 18;6(12):5900–14. 10.3390/nu6125900 25529062 PMC4277006

[24] Kennedy D, Koren G. Identifying women who might benefit from higher doses of folic acid in pregnancy. Can Fam Physician. 2012;58: 394–397. 22499814 PMC3325450

[25] LaMarca BD, Gilbert J, Granger JP. Recent Progress Toward the Understanding of the Pathophysiology of Hypertension During Preeclampsia. Hypertension. 2008;51: 982–988. 10.1161/HYPERTENSIONAHA.107.108837 18259004 PMC2782443

[26] Daly S, Cotter A, Molloy AE, Scott J. Hcy and folic acid: implications for pregnancy. Semin Vasc Med. 2005; 5:190–200. 16047271 10.1055/s-2005-872404

[27] Catov JM, Nohr EA, Bodnar LM, Knudson VK, Olsen SF, Olsen J. Association of periconceptional multivitamin use with reduced risk of preeclampsia among normal-weight women in the Danish National Birth Cohort. Am J Epidemiol. 2009;169: 1304–1311. 10.1093/aje/kwp052 19372217 PMC2727249

[28] Pittschieler S, Brezinka C, Jahn B, Trinka E, Unterberger I, Dobesberger J, et al Spontaneous abortion and the prophylactic effect of folic acid supplementation in epileptic women undergoing antiepileptic therapy. J Neurol. 2008; 255(12):1926–31. 10.1007/s00415-008-0029-1 18677647

[29] Czeizel AE, Bánhidy F. Folic acid supplementation and risk reduction in preterm birth. Am J Clin Nutr. 2011;94(6):1651–2. 10.3945/ajcn.111.026690 22106418

[30] Timmermans S, Jaddoe VW, Hofman A, Steegers-Theunissen RP, Steegers EA. Periconception folic acid supplementation, fetal growth and the risks of low birth weight and preterm birth: the Generation R Study. Br J Nutr. 2009;102(5):777–85. 10.1017/S0007114509288994 19327193

[31] Wang J, Ge J, Yang LN, Xue D, Li J. Protective effects and its mechanism on neural cells after folic acid intervention in pre-eclampsiarat model. Zhonghua Fu Chan Ke Za Zhi. 2011;46: 605–609. 22169520

[32] Mujawar Shahid A., Patil Vinayak W., and Daver Rekha G.. study of plasma hemocystein, folic acid and vitamin B12 in patients with pre-eclmpsia. Indian J Clin Biochem. 2011; 26: 257–260. 10.1007/s12291-011-0109-3 22754189 PMC3162959

[33] Czeizel AE, Puhó EH, Langmar Z, Acs N, Bánhidy F. Possible association of folic acid supplementation during pregnancy with reduction of preterm birth: a population-based study. Eur J Obstet Gynecol Reprod Biol. 2010;148: 135–140. 10.1016/j.ejogrb.2009.10.016 19926391

[34] Acilmis YG, Dikensoy E, Kutlar AI, Balat O, Cebesoy FB, Ozturk E, et al Hcy, folic acid and vitamin B12 levels in maternal and umbilical cord plasma and Hcy levels in placenta in pregnant women with pre-eclampsia. J Obstet Gynaecol Res. 2011;37: 45–50. 10.1111/j.1447-0756.2010.01317.x 21040211

[35] Salehi-PourMehr H, Mohamad-Alizadeh S, Malakouti J, Farshbaf-Khalili A.Association of the folic acid consumption and its serum levels with preeclampsia in pregnant women.Iran J Nurs Midwifery Res. 2012; 17(6): 461–466. 23922590 PMC3733294

[36] Qin X, Cui Y, Shen L, Sun N, Zhang Y, Li J, et al Folic acid supplementation and cancer risk: a meta-analysis of randomized controlled trials. Int J Cancer. 2013;133: 1033–1041. 10.1002/ijc.28038 23338728

[37] Taylor CM, Atkinson C, Penfold C, Bhattacharya S, Campbell D, Davey Smith G, et al Folic acid in pregnancy and mortality from cancer and cardiovascular disease: further follow-up of the Aberdeen folic acid supplementation trial. J Epidemiol Community Health. 2015; 69(8):789–94. 10.1136/jech-2014-205324 25855124 PMC4515996

[38] Vollset SE, Clarke R, Lewington S, Ebbing M, Halsey J, Lonn E, Armitage et al Effects of folic acid on overall and site-specific cancer incidence during the randomised trials: meta-analyses of data on 50 000 individuals Lancet. 2013; 23;381(9871):1029–36. 23352552 10.1016/S0140-6736(12)62001-7 PMC3836669

[39] Piyathilake CJ, Macaluso M, Chambers MM, Badiga S, Siddiqui NR, Bell WC, et al Folate and vitamin B12 may play a critical role in lowering the HPV 16 methylation-associated risk of developing higher grades of CIN.Cancer Prev Res (Phila). 2014;7(11):1128–37. 25145486 10.1158/1940-6207.CAPR-14-0143 PMC4711818

