# Supplementary material for: The Effect of High Dose Folic Acid throughout Pregnancy on Homocysteine (Hcy) Concentration and Pre-Eclampsia: A Randomized Clinical Trial
Source: PLoS One. 2016 May 11;11(5):e0154400. doi: 10.1371/journal.pone.0154400 (PMC4868051; doi:10.1371/journal.pone.0154400)
Supplement: S1 Appendix — (DOCX) [file pone.0154400.s001.docx]

**Clinical trial protocol**

IRCT registration number: IRCT registration number: IRCT201402175283N9

Date registered: March 25, 2014

Registration timing: Retrospective registration

Recruitment status: Recruitment complete

Expected recruitment start date: 2006-08-22

Expected recruitment end date: 2013-08-23

Scientific title: The effect of prenatal administration of high dose and low dose folic acid on maternal plasma homocysteine concentration and its relationship with preeclampsia

Summary:

OBJECTIVE: To evaluate effects of high and low dose folic acid on levels of homocysteine (Hcy) concentration during first trimester of pregnancy and at delivery, and to examine the association of Hcy serum levels and preeclampsia. METHODS: A single blinded randomized clinical trial will be conducted in Tabriz, Iran, from 2006-2013, in 410 nulliparous pregnant women in 2 groups, who will receive folic acid daily from early pregnancy until delivery (5 mg/day and 0.5 mg/ day respectively). The incidence of pregnancy induced hypertension, preeclampsia, eclampsia and laboratory changes in the levels of serum Hcy, Platelet numbers, lactate dehydrogenase, uric acid, serum and urine creatinine and serum and urine protein will be compared. Maternal blood pressure and weight will be checked in each visit including labor time. The number of abortions, premature labor, IUGR, fetal death, PIH, preeclampsia, eclampsia, birth weight, congenital abnormalities, andthe date of pregnancy termination will be reported in a questionnaire. Healthy mothers with a singleton pregnancy aged between 20-30 years old were included and patients with heart disease; chronic hypertension; diabetes mellitus; collagen vascular diseases; chronic renal disease; and who taking medications other than calcium and ferrous sulfate and vitamin B6 will be excluded.

Public title: The effect of perinatal administration of folic acid on maternal plasma homocysteine concentration and its relationship with the development of preeclampsia

Purpose: Prevention

Inclusion/exclusion criteria: Inclusion criteria: Healthy mothers with a singleton pregnancy aged between 20-30 years old Exclusion criteria: Heart disease; chronic hypertension; Diabetes mellitus; collagen vascular diseases; chronic renal disease; preterm delivery for the other reasons except preeclampsia; taking medications other than calcium and ferrous sulfate and vitamin B6.

Minimum age: Year: 20

Maximum age: Year: 30

Gender: female

Health condition table is empty1: preeclampsia

Condition studied: O14.9

Condition ICD-10 code : Gestational [pregnancy-induced] hypertension with significant proteinuria

Intervention/Control1 Control group: folic acid 0.5mg/day from the beginning of the pregnancy to the end.

Intervention category Treatment: drugs

Intervention/Control2 Intervention group: folic acid 5mg/day from the beginning of the pregnancy to the end.

Intervention category Treatment: drugs

Primary outcome measure1: Decreasing in homocysteine level

Primary outcome time point: first trimester and at delivery time

Primary outcome method of measurement: serum sampling

Primary outcome measure2: prevention of preeclampsia

Primary outcome time point: after 20th week of pregnancy age

Primary outcome method of measurement: controlling of severe signs, body weight, blood pressure, edema, urine protein in every visit

Secondary outcome measure 1 : preterm labor

Secondary outcome time point: labor after 20-37

Secondary outcome method of measurement: clinical examination

Secondary outcome measure 2: Prevention of IUGR

Secondary outcome time point: After 24 weeks every month

Secondary outcome method of measurement: sonography

Secondary outcome measure 3:1- and 5-minute Apgar scores

Trial phase : [N/A].
